# Normative influences on intentions to smoke among Greek adolescents: the moderating role of smoking status

**DOI:** 10.1186/1617-9625-12-5

**Published:** 2014-03-26

**Authors:** Lambros Lazuras

**Affiliations:** 1 Psychology Department, International Faculty of the University of Sheffield, CITY College, Thessaloniki, Greece; 2 South-East European Research Centre (SEERC), Thessaloniki, Greece

**Keywords:** Adolescents, Social norms, Smoking intentions

## Abstract

**Background:**

Social norms influence adolescent smoking intentions, but this effect may differentiate depending on current smoking experiences. The presented study assessed the moderation effects of smoking status on the relationship between social norms and smoking intentions among Greek adolescents.

**Methods:**

A cross-section survey-based design was used. Overall, 251 Greek secondary school students (M age = 16.1 years, 61.2% females) completed structured and anonymous questionnaires including demographic characteristics (age, gender), subjective and descriptive social norms towards smoking, self-reported tobacco use, and intentions to smoke in the next 12 months.

**Results:**

Linear regression analysis showed that social norms overall predicted 36.4% (Adjusted *R*^2^) of the variance in intentions. Perceived prevalence of smoking in same age peers and adults, having more close friends who smoke and perceived social approval of smoking predicted intentions to smoke in one year. Moderated regression analysis showed that the effects of social norms on smoking intentions were significantly moderated by smoking status.

**Conclusions:**

Social norms predict smoking intentions, but this effect is stronger among ever (than never) smoker adolescents. Adolescents with smoking experiences may selectively attend to pro-smoking social cues and this perpetuates into their motivation to keep up the habit. School-based interventions should target normative beliefs and related cognitive processes, especially among adolescents who have already initiated tobacco use.

## Background

Tobacco use is the leading cause of preventable death in the world, causing about 5 million deaths annually, and most adult smokers take up the habit during adolescence [1,2]. Indeed, adolescence is described as the life period where health risk behaviors are frequently enacted, especially in the presence of same age peers, spanning from careless driving to tobacco and drug use [3,4]. Attendance to social norms and peer influence are among the most prominent explanations for adolescent health risk-taking. Adolescents respond differently than adults to peer presence as indicated by activation in the socio-emotional areas [5,6]. This process makes adolescents prone to take more risks when peers are present, but less risks in the absence of their peers [7,8].

Social norms represent people’s worldviews in relation to what is seen as socially acceptable (subjective or injunctive norms), or popular and prevalent (descriptive norms) [9]. Research has consistently shown that stronger pro-smoking norms are related to stronger intentions to take up or continue smoking during adolescence [10-13]. It is also well established that tobacco use experimentation in teenage years predicts future smoking trends in late adolescence and adulthood [14]. What is less clear is how smoking experiences attenuate or intensify the effects of social norms on intentions to smoke. That is, peer influences and other types of social norms might be more influential on decisions to continue smoking among early experimenter smokers, than on the decision to take up smoking among non-smoker adolescents. In short, social norms may differentially influence smoking decisions among adolescents, depending on their current habits.

The theoretical basis for this argument comes from research in social and health psychology which shows that people tend to see the world in a self-justifying way in order to relieve the tension that may occur from the incongruence between belief systems (e.g., attitudes, social norms) and actual behavior [15]. So, compared to non-smokers, smokers tend to downplay the health risks of tobacco use [16,17], perceive smoking as more prevalent and socially acceptable than it actually is [18,19], and strongly oppose tobacco control policies as such policies would directly undermine their current habits [20,21]. In a similar vein, adolescents who currently smoke may perceive smoking as more socially acceptable and prevalent than non-smokers, and this may influence their intentions to continue smoking [22].

The present study aims to address the role of smoking experiences in the relationship between social norms and intentions to smoke among Greek adolescents. Although Greece is among the economically developed countries of the world and there is recent evidence about the success of tobacco control policies, still smoking prevalence rates are very similar to that of developing countries [23,24]. Thus, Greek adolescents grow up in an environment where the prevailing social norms are still pro-smoking, tobacco control policies are weakly enforced, and exposure to family smoking and secondhand smoke is widely prevalent [25,26]. Accordingly, pro-smoking social norms among Greek adolescents have been associated with stronger intentions to smoke and weaker self-efficacy to resist smoking in risk-conducive situations, such as peer pressure [22]. The present study goes beyond this association, and assesses if pro-smoking social norms predict intentions differently between smoker and non-smoker adolescents. It is hypothesized that social norms will predict smoking intentions more strongly among adolescents with smoking experiences, as compared to non-smoker adolescents.

## Methods

### Study design and sample

A cross-sectional, survey-based design was used. Overall, structured anonymous questionnaires were given to 300 adolescent secondary school students, and 251 completed questionnaires were returned (response rate = 83.6%). Participants were aged between 14 to 18 years (*M* age = 16.11 years, *SD* = 0.89), and 61.2% (*n* = 153) were females. Ethics approval for this study was granted by the Ethics Review Board of the International Faculty of the University of Sheffield, and informed consent was obtained from participants’ parents/caregivers.

### Measures

Most of the measures in the questionnaire were used in previous studies with Greek adolescents [21,22]. Age and gender were assessed with single items. Smoking status was also measured with a single item (*‘Have you ever smoked?’*) followed by fie response options (1 = no, I never tried smoking; 2 = yes, I have smoked some times, but less than 5 cigarettes overall; 3 = yes, I smoke occasionally in the week, but not on a daily basis; 4 = yes, I smoke at least one cigarette a day; and 5 = I used to smoke in the past, but I have given up). For purposes of subsequent analysis, two groups were developed by collapsing categories of smoking status. Specifically, respondents who reported that they never tried smoking were classified as ‘never smokers’ and those reporting past or current smoking status were classified as ‘ever smokers’.

Social norms were measures with respect to subjective (i.e., perceived social approval of smoking by referent others) and descriptive normative beliefs (i.e., perceived and current smoking prevalence in the peer group, and exposure to smoking at home and in public places). Subjective norms were measured with the mean score of three items on the perceived approval of smoking by young adults (18-25 years old), adults (>25 years old), and parents. Responses were recorded on a 7-point continuous scale, 1 = strongly disagree to 7 = strongly agree. Internal consistency reliability was high (Cronbach’s *α* = 0.85), and higher mean scores in this variable reflected greater disapproval of smoking.

Perceived prevalence of smoking among same age peers and adults in the country was assessed with two single items respectively asking participants to give a percentage estimate (from 0 to 100%) reflecting how many same age peers and adults are smokers (smoking at least one cigarette a day). Peer group smoking was assessed with a single item asking participants to indicate the number of smokers among their five closest friends. Exposure to family smoking was assessed with the question ‘how often do you see family members smoking at home’, and responses were scored on five-point continuous scale, 1 = never to 5 = always. Exposure to public smoking was assessed with the item *‘how often do you see each of the following groups smoking in public or open places, such as cafeterias, restaurants, school premises, and bus stations?’*. Responses were recorded for same age peers, young adults (18-25 years old), and adults (>25 years) on a five-point continuous scale, 1 = never to 5 = always. A mean score was computed (Cronbach’s *α* = .70), and higher scores reflected more frequent exposure to public smoking.

Smoking intentions were assessed with the mean of three items using a 12 month time frame (e.g., *‘do you think you will be smoking in the next year’*), and responses were recorded on a 7-point continuous scale, 1 = definitely not to 7 = definitely yes. Internal consistency reliability was high (Cronbach’s *α* = .96), and higher scores reflected stronger intentions to smoke in the next year.

### Data analysis

Analysis of frequencies with chi-square (*χ*^2^) was used to assess prevalence rates of smoking and gender differences in self-reported smoking status. One-way ANOVA was used to assess differences in age, as well as differences in social normative beliefs and smoking intentions between never and ever smokers. Linear regression analysis was used to assess the direct effects of subjective and descriptive social norms on smoking intentions. Standardized beta weights (β) are used to denote the predictive effects of social norms on intentions. Finally, moderated regression analysis was used to assess the moderating effects of smoking status on the relationship between social norms and smoking intentions. Significance level for all analyses was set at *p* < 0.05.

## Results

### Smoking status: prevalence, gender and age differences

Roughly half (50.8% or *n* = 127) of the participants were never smokers, whereas 21.6% (*n* = 54), reported they have smoked less than 5 cigarettes in their lifetime, 8% (*n* = 20) reported they were occasional/weekly smokers, 11.2% (*n* = 28) were daily smokers, and 8.4% (*n* = 21) said they used to smoke but have given up at the time the survey was completed. The ANOVA results did not show significant age differences between ever and never smokers. Accordingly, chi-square analysis of frequencies did not show statistically significant gender differences in self-reported smoking status.

### Differences in social normative beliefs and intentions by smoking status

One-way ANOVA showed that, compared to never smokers, smoker adolescents reported stronger intention to smoke in the next 12 months (*F* = 65.69, *p* < .001, *η*^2^ = 0.20), had more closest friends who smoked (*F* = 61.99, *p* < .001, *η*^2^ = 0.20), were more frequently exposed to public smoking (*F* = 14.13, *p* < .001, *η*^2^ = 0.05), viewed smoking as more prevalent among same-age peers in the country (*F* = 20.57, *p* < .001, *η*^2^ = 0.07), and perceived less social disapproval of smoking (*F* = 6.82, *p* = .01, *η*^2^ = 0.02). Effect sizes were moderate to strong according to [27]. The findings are summarized in Table 1.

**Table 1:** Differences in smoking intentions and social norms between ever and never smokers

		**Never smokers**		**Ever smokers**	
	**F**	***M***	***SD***	***M***	***SD***
Intentions to smoke in 12 months	65.69**	1.20	0.76	2.79	2.00
Number of close friends who smoke	61.99**	1.86	1.26	3.30	1.61
Public smoking exposure	14.13**	3.90	0.55	4.16	0.55
Perceived prevalence of smoking in same age peers	20.57**	43.19	21.85	55.49	20.58
Subjective norms	6.82*	6.20	1.33	5.74	1.47

Note. *p < .05, **p < .005.

### Direct effects of social norms on smoking intentions

Linear regression analysis was used to assess the direct effects of descriptive and subjective social norms on smoking intentions. Overall, a significant model emerged predicting 36.4% of the variance in smoking intentions (*F* = 17.84, *p* < .001). Significant predictors of smoking intentions included number of close friends who smoke (*β* = .502, *p* < .001), perceived prevalence of smoking among adolescents (*β* = .177, *p* = .005) and adults (*β* = −.169, *p* = .006) in Greece, and subjective norms (*β* = −.144, *p* = .008). The findings from the regression analysis are presented in Table 2.

**Table 2:** Effects of social norms on smoking intentions

**Predictors**	**95% CI**	***β***	**Adj** ***R***^**2**^
Age	−.081 to .327	.063	.364
Gender	−.473 to .256	−.031	
Number of closest friends smoking	.414 to .653	.502**	
Exposure to family smoking	−.097 to .138	.018	
Exposure to public smoking	−.320 to -.353	.005	
Perceived prevalence of smoking (peers)	.004 to .024	.177*	
Perceived prevalence of smoking (adults)	−.026 to -.004	−.169*	
Subjective norms	−.312 to −.048	−.144*	

Note. *p < .05, **p < .005.

### Interaction between smoking status, social norms and intentions to smoke

**Figure 1:**
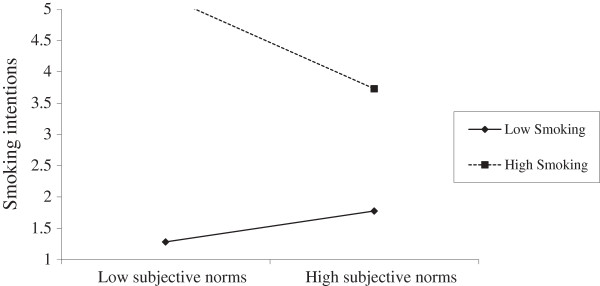
Interaction between smoking status and subjective norms.

**Figure 2:**
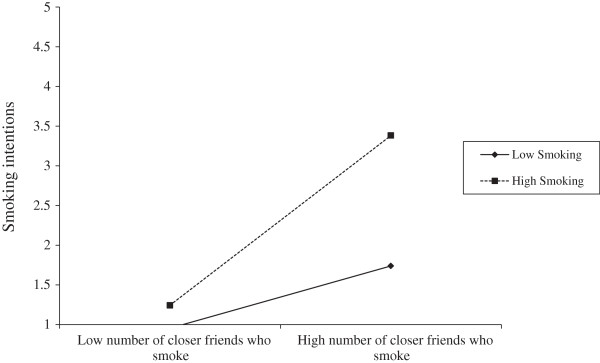
Interaction between smoking status and number of close friends who smoke.

Three moderated regression analyses were used to respectively assess the interaction of smoking status with number of smoker close friends, perceived prevalence of adolescent smoking in Greece, and subjective social norms (acceptability of smoking). Following the recommendations by Aiken and West [28], the predictor variables were mean centered in order to avoid multicollinearity, and an interaction terms (social norm × smoking intention) was computed to for each measure of social norms. Unstandardized coefficients (Β) were used to denote the weight of the interaction effect, and 95% confidence intervals (CIs) were computed. The results showed that smoking significantly moderated the effects of social norms on smoking intentions with respect to number of close friends who smoke (B _N of close friends smoking × smoking status_ = .421, *p* = .001, 95% CI = .184 to .657) and subjective norms (B _subjective norms × smoking status_ = −.494, *p* < .001, 95% CI = −.756 to −.233). In the case of perceived prevalence of smoking among same age peers, although the interaction term was marginally significant (B _perceived prevalence of smoking × smoking status_ = .019, *p* = .046), the 95% CIs were in the range of zero (.000 to .037), thus the interaction was deemed non-significant. In short, number of close friends and subjective norms were more influential on smoking intentions among current smokers, than never smoker adolescents. Simple slope analyses for each significant interaction effect are respectively presented in Figures 1 and 2.

## Discussion

The present study set out to assess the direct effects of social normative beliefs, specifically subjective (perceived social approval of smoking) and descriptive norms (perceived prevalence and exposure to smoking cues) on adolescents’ intentions to smoke. The role of smoking status was highlighted in this relationship, mainly in terms of moderating normative influences on smoking intentions. It was expected that both descriptive and subjective social norms would predict smoking intentions, and that normative influences on intentions would be stronger among ever than never smokers. The results from the linear regression analysis showed that number of close friends, perceived prevalence of smoking among same age peers and adults in the country, and subjective norms significantly predicted intentions to smoke in 12 months. Exposure to public and family smoking did not have a significant effect, thus partially supporting the study’s initial hypothesis. With respect to the moderating effect of smoking status, moderated regression analysis showed that the effects of subjective norms and number of close friends who smoke on intentions was stronger among ever than never smokers, thus confirming the study’s hypothesis.

The aforementioned findings highlight the important role of smoking status on normative influences on adolescent smoking. Specifically, perceived norms about smoking seem to be more influential among adolescents who already experimented with (or currently engage in) tobacco use, than among adolescents without smoking experiences. One way of explaining these findings relates to the cognitive biases that are typical among smokers, such as the tendency to misperceive the prevalence or social acceptability of smoking in peer groups [29,30]. Another explanation is that smokers are more likely to be exposed to smoking-conducive situations, thus, their perceptions of social norms are shaped by their experiences in smoking (than non-smoking situations). Besides, the findings from the ANOVA showed that ever smokers perceived smoking as more acceptable and prevalent, and were more frequently exposed to public smoking than never smokers. Thirdly, it may be the case that smoker adolescents display memory recall bias by recalling more easily pro-smoking, than anti-smoking, social norms, and this provides them with the means to self-justify for their behavior and future intentions. Related arguments about attentional biases in the context of substance use are presented by Field and Cox [31]. All these explanations are complementary and not mutually exclusive, and they point to the profound need to alleviate potential tension or incongruence between beliefs and actions. In this respect, behavior seems to be ‘the horse’ and social norms are ‘the cart’. Nevertheless, although this assumption rests in well established findings in social and health psychology [15,32], more studies are needed to establish causality.

Furthermore, the present study was in line with past research among Greek adolescents [22] showing that subjective and descriptive social norms play an important role in the decision to smoke. Nevertheless, the non-significant effect of family and public smoking exposure is in contrast with past studies [33,34]. One possible explanation is that, when controlling for more immediate and powerful sources of normative influence, such as peer group or close friends’ smoking [10], the effects of more ‘distal’ normative influences, such as public and family smoking exposure become attenuated and turn up to be non-significant. Accordingly, it is noteworthy that although perceived prevalence of smoking among adults had a significant direct effect on adolescents’ smoking intentions, this effect was negative: the higher the perceived prevalence of smoking in adults, the lower the intention to smoke in adolescents. This is an interesting finding that comes in opposite direction to the effect of perceived prevalence of smoking among same age peers on intentions. Interestingly, one explanation pertains to social distance (i.e., perceived similarity to a target group being evaluated) [35,36]. That is, adolescents may be more likely to attend to smoking norms among referent peer groups, than among non-referent adult groups. Similarity is greater, and hence social distance is smaller, among same age peers, whereas the reverse pattern is true for adults.

The key point of the present findings for policy makers is that there may be a need for a paradigm shift in the way social norms are viewed as influences on adolescent smoking. Although we do not claim that social norms cannot drive or shape adolescents’ smoking tendencies, we do call for greater attention to cognitive biases and the need to maintain consistency between actions and belief systems. Adolescent smokers may simply be more motivated to recall pro-smoking norms as a way of self-justifying their current behavior. If this is true, then intervention targeting current or ever smoker adolescents should not only focus on making pro-smoking norms less salient, they should also target the very cognitive mechanisms that give rise to self-serving biases.

## Competing interests

The author declares that he has no competing interests.
